# Formation mechanism of fivefold deformation twins in a face-centered cubic alloy

**DOI:** 10.1038/srep45405

**Published:** 2017-03-28

**Authors:** Zhenyu Zhang, Siling Huang, Leilei Chen, Zhanwei Zhu, Dongming Guo

**Affiliations:** 1 Key Laboratory for Precision and Non-Traditional Machining Technology of Ministry of Education, Dalian University of Technology, Dalian 116024, China; 2 Key laboratory of Marine Materials and Related Technologies, Ningbo Institute of Materials Technology and Engineering, Chinese Academy of Sciences, Ningbo 315201, China; 3 Changzhou Institute of Dalian University of Technology, Changzhou 213164, China

## Abstract

The formation mechanism considers fivefold deformation twins originating from the grain boundaries in a nanocrystalline material, resulting in that fivefold deformation twins derived from a single crystal have not been reported by molecular dynamics simulations. In this study, fivefold deformation twins are observed in a single crystal of face-centered cubic (fcc) alloy. A new formation mechanism is proposed for fivefold deformation twins in a single crystal. A partial dislocation is emitted from the incoherent twin boundaries (ITBs) with high energy, generating a stacking fault along {111} plane, and resulting in the nucleating and growing of a twin by the successive emission of partials. A node is fixed at the intersecting center of the four different slip {111} planes. With increasing stress under the indentation, ITBs come into being close to the node, leading to the emission of a partial from the node. This generates a stacking fault along a {111} plane, nucleating and growing a twin by the continuous emission of the partials. This process repeats until the formation of fivefold deformation twins.

Nanocrystalline (nc) metals are widely used in consumer electronics, biosensing, nanomedicine, optical systems, drug delivery, photovoltaic cells and organic solar cells, owing to their mechanical and optoelectronic properties^1^,^2^,^3^,^4^. Fivefold twins (FTs) increase the yield strength and elastic modulus of the nc metals, compared to their monocrystalline counterparts, attracting much attentions^5^,^6^,^7^,^8^. FTs play an important role to improve the mechanical properties of nc metals. Nevertheless, FTs are usually observed in grains of nanowires, nanoparticles and nc metals^9^, whereas rarely found in single crystals and macroscopic materials, except occasionally in highly deformed metals, such as ball milling and high pressure torsion^9^,^10^,^11^. This raises an issue for the formation mechanism of fivefold deformation twins in the single crystals and macroscopic materials for pragmatic engineering applications.

At present, the work of FTs mainly focuses on face-centered cubic (fcc) single elemental metals, such as copper (Cu)^9^,^12^,^13^, silver (Ag)^4^, gold (Au)^1^, aluminum (Al)^14^, nickel (Ni)^10^,^15^ and iron (Fe)^6^. Ball milling and high pressure torsion are used to fabricate nc metals by plastic deformation, in which FTs are occasionally observed^9^. Molecular dynamics (MD) is an effective method to investigate the formation mechanism of fivefold deformation twins. Nevertheless, MD simulations mainly concentrate on the nc single elemental metals^5^,^6^,^7^,^8^,^10^,^12^,^14^,^16^,^17^. Engineering materials are primarily alloys, rather than single elemental metals. Moreover, MD simulations dominate nc^7^,^8^,^10^,^12^,^14^,^17^ and constructed models^5^,^6^,^16^, other than a single crystal. This results in the interpretation that formation of FTs, is through the emission of partial dislocations from the grain boundaries (GBs)^5^,^6^,^7^,^8^,^10^,^12^,^14^,^16^,^17^. Therefore, fivefold deformation twins derived from a single crystal have not been reported by MD simulations. For an alloy, the sizes of grains are normally several and tens of micrometers, which are two and three orders magnitude higher than those of FTs in nc metals. Hence, the size of a grain in an alloy is similar to a single crystal for an FT generated in an nc metal. Because there are no GBs in a single crystal, the formation mechanism of fivefold deformation twins in a single crystal is different from an nc metal.

In this study, fivefold deformation twinning is performed by MD simulations in a single crystal of a Haynes alloy of the C-2000^®^ with an fcc structure. The formation mechanism of fivefold deformation twins is investigated in a single crystal under nanoindentation via plastic deformation.

## Results

**Figure 1:**
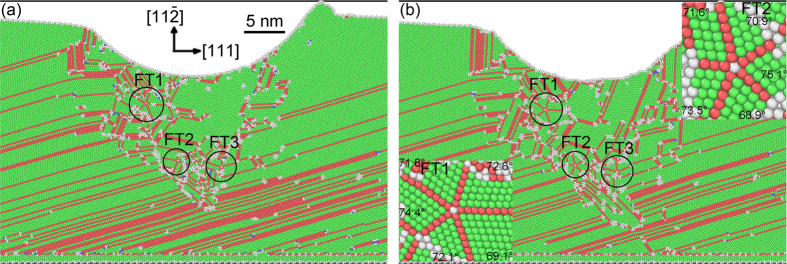
Snapshots of fivefold deformation twinning at an indentation depth of (**a**) 9 nm and (**b**) after unloading. Insets in (**b**) showing its corresponding enlarged FTs.

Figure 1 illustrates the snapshots of fivefold deformation twinning at an indentation depth of 9 nm and after unloading. Three FTs are observed in Fig. 1(a), and marked by three black circles. The three FTs look better after unloading compared to those in Fig. 1(a), as shown in Fig. 1(b). One enlarged FT at the lower left in Fig. 1(b) has angles of 69.1°, 72.6°, 71.8°, 74.4°, 72.1°, and the other at the upper right has 68.9°, 75.1°, 70.9°, 71.6°, and 73.5°.

**Figure 2:**
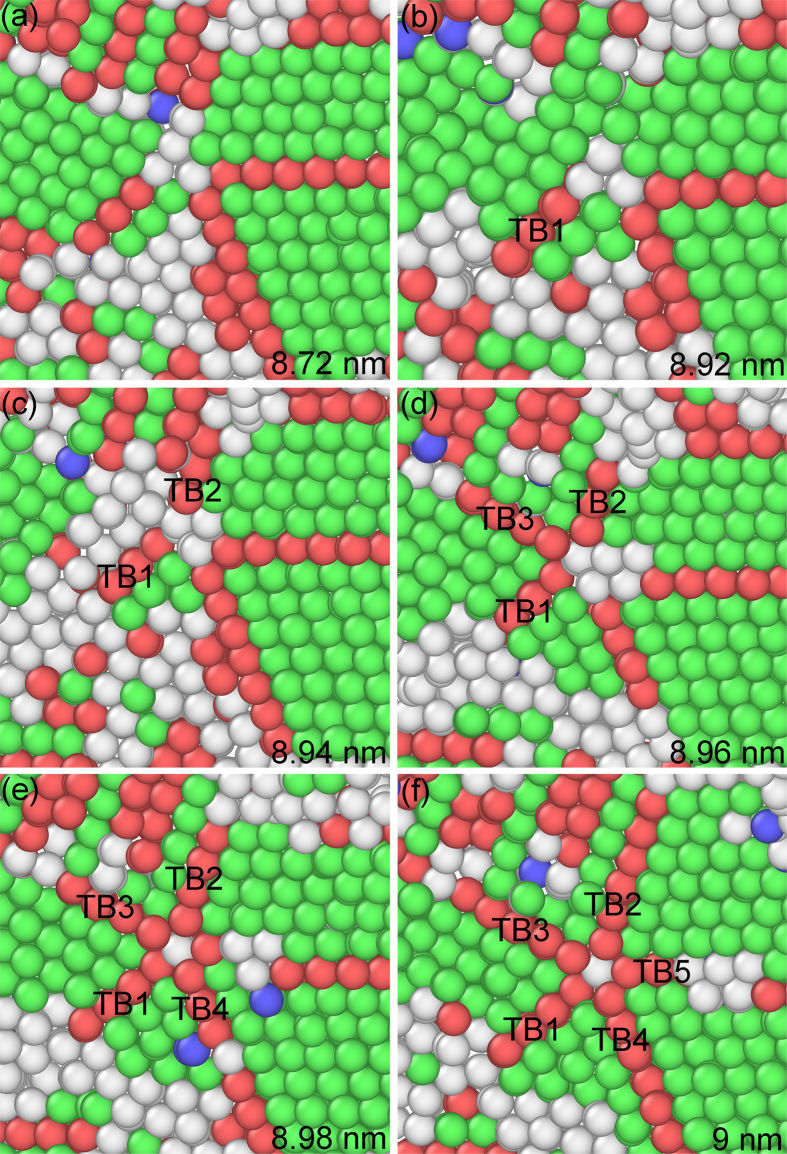
Snapshots of formation on a fivefold deformation twin marked by FT1 in Fig. 1(a) at indentation depths of (**a**) 8.72, (**b**) 8.92, (**c**) 8.94, (**d**) 8.96, (**e**) 8.98 and (**f**) 9 nm.

Figure 2 shows the snapshots of formation on a fivefold deformation twin marked by FT1 in Fig. 1(a) at different indentation depths. An intersecting center is formed in Fig. 2(a) among four different slip {111} planes. Twin boundary (TB) marked by TB1 is produced by white atoms, as illustrated in Fig. 2(b). TB2 comes into being close to the intersecting point of the four different slip planes of {111}, forming a node between TB1 and TB2. TB3 is emitted from the node with increasing stress induced by indentation (Fig. 2(d)). TB4 is emitted from the node, connecting with the existing TB (Fig. 2(e)). TB5 is emitted from the node with increasing stress, creating a fivefold deformation twin (Fig. 2(f)).

**Figure 3:**
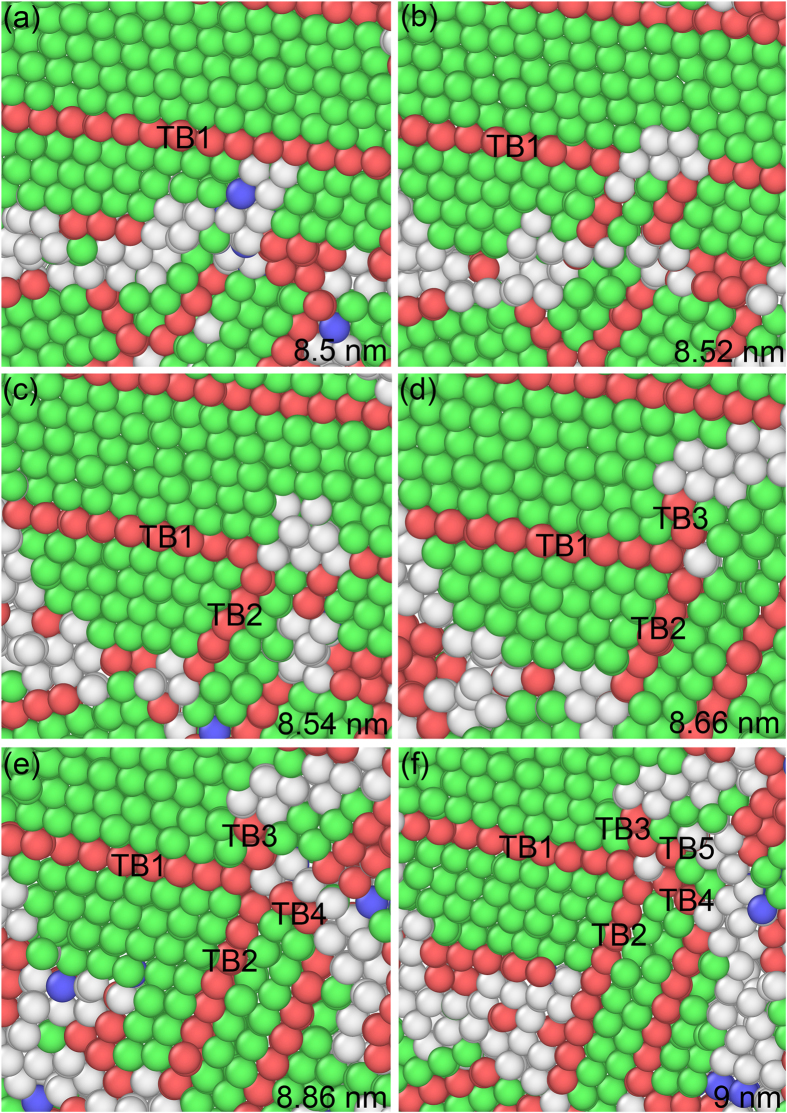
Snapshots of formation on a fivefold deformation twin marked by FT2 in Fig. 1(a) at indentation depths of (**a**) 8.5, (**b**) 8.52, (**c**) 8.54, (**d**) 8.66, (**e**) 8.86 and (**f**) 9 nm.

Figure 3 depicts the snapshots of formation on a fivefold deformation twin marked by FT2 in Fig. 1(a) at different indentation depths. Incoherent twin boundaries (ITBs) are observed in Fig. 3(a) displayed by white atoms. A TB is formed by white atoms, as shown in Fig. 3(b), which is the origin of TB2 (Fig. 3(c)). With increasing indentation depth, TB2 is generated in Fig. 3(c) forming a node between TB1 and TB2. TB3 is emitted from the node, as illustrated in Fig. 3(d). Increasing indentation depth, TB4 is formed from the node (Fig. 3(e)). Finally, TB5 is generated from the node (Fig. 3(f)) creating a fivefold deformation twin.

## Discussion

Fivefold deformation twins derived from a single crystal are not predicted by MD simulations. This is attributed to that the formation mechanism of fivefold deformation twins explains, that a simple twin is generated through partial dislocation emissions from GBs^5^,^6^,^7^,^8^,^10^,^12^,^14^,^16^,^17^, resulting in the fivefold deformation twins observed in nc metals^7^,^8^,^10^,^12^,^14^,^17^. Furthermore, it is reported that the formation mechanism of fivefold deformation twins requires an orientation change of applied stresses, leading to the absence of fivefold deformation twins in MD simulations induced by a uniaxial stress^9^. However, fivefold deformation twins are observed in our MD simulations derived from a single crystal under nanoindentation. This is different from previous literatures^5^,^6^,^7^,^8^,^10^,^12^,^14^,^16^,^17^, in which fivefold deformation twins are found in nc metals. Wherefore, it is intriguing to elucidate the formation mechanism of fivefold deformation twins in a single crystal under nanoindentation.

The C-2000^®^ has an fcc structure with a low stacking fault energy of 1.22 mJ m^−2^ 18. Although indentation is loaded at the [-1-12] orientation on the single crystal, there are (111), (-111), (1-11) and (11-1) four different slip planes for an fcc structure in the single crystal. The Schmid factors are 0.416, 0.416, 0.314, and 0 for four slip systems [-1-12](-111), [-1-12](1-11), [-1-12](11-1), and [-1-12](111), respectively. The [-1-12](-111) and [-1-12](1-11) slip systems have the highest Schmid factor, meaning the highest shear stress and becoming the optimal slip planes. At the intersecting area of different slip orientations, plenty of ITBs are formed, which is displayed by white atoms, as illustrated in Fig. 1. For an alloy, it is difficult to find the energetic values from literatures consisting of ITBs, coherent TBs (CTBs), and grain boundaries. However, for a pure metal, it is relatively easy to obtain these values. Ni element occupies the 61% weight percentage of the ternary alloy used in the MD simulations, and Ni crystal has an fcc structure that is consistent with the alloy. Therefore, Ni is used to present the difference among distinct energies. For a pure Ni, the stacking fault energy is 125 mJ m^−2^, and its CTB energy is 63 mJ m^−2^ 10. For a comparison, the general GB of Ni has an energy of 1469 mJ m^−2^, which is an order magnitude higher than CTB^10^. However, an ITB has an energy much closer to a general GB, rather than a CTB in Ni. To relax the stress concentration and store the energy, lots of ITBs are observed in Fig. 1, which are displayed by white atoms. ITBs are the products of collided slip orientations among the four different slip planes, and are stacking faults in nature with high energy. Stress concentrates at the ITBs, relieving the energy barrier to emit partial dislocations. Many ITBs play an important role to form fivefold deformation twins, which is similar to grain boundaries^9^,^13^. Nonetheless, ITBs are different from the disordered GBs. The white atoms are essentially crystals with high energy, and can transform into perfect atoms, twins and stacking faults under stress. The more stress concentration is, the more white atoms are, as depicted in Fig. 1. The highest stress concentration takes place at the intersecting center of four different slip {111} planes, resulting in the formation of fivefold deformation twins in Fig. 1 and Fig. 2(a). At the intersecting center, white atoms have high energy, as illustrated in Fig. 1(a) by FT1, FT2 and FT3. A partial dislocation is emitted from the white atoms by increasing stress induced by indentation. This is because of the white atoms with high energy lowering the energy barrier for partial emission. Then, a stacking fault is generated along a {111} plane by the first partial from the white atoms, resulting in a twin nucleating and growing via successive emission of the partials from the white atoms by the increasing stress, as shown in Figs 2(b) and 3(b). With the growing of twins, TB1 and TB2 are generated in Figs 2(b) and 3(c), respectively. A white atom at the intersecting center among the four different slip planes was fixed and became a node, as shown in Figs 2(c) and 3(c). The stress concentrated at the node with increase stress loaded by indentation, leading to the highest energy stored and stress concentration at the local area. To release the stress and high energy stored at the node, ITBs displayed by white atoms come into being close to the node along {111} slip orientations, as illustrated in Figs 2(c) and 3(c). With increasing stress under indentation, the node has the driving force to emit the partial dislocation, due to its high energy lowering the energy barrier for the emission of a partial. As the angle between two different {111} slip orientations is 70.53° in an fcc crystal, stacking faults generated by the partial emitted from the node glided along the orientation with an angle of 70.53° or 141.06° away a twin. This enabled the nucleation and growth of a twin with the successive emission of the partials induced by stress under indentation, resulting in the formations of TB2 and TB3 in Figs 2(c) and 3(d), respectively. Increasing the indentation depth, ITBs generated close to the node to release the stress and high energy at the node, emitting a partial from the node. This creates a stacking fault along a {111} plane, nucleating and growing a twin by continuous emission of the partials from the node by increasing stress under indentation, then forming TB3 and TB4 in Figs 2(d) and 3(e), respectively. The formations of threefold and fourfold deformation twins store high energy, resulting in the release of stress stored by the white atoms. This makes the white atoms transform into green perfect atoms, as illustrated in Figs 2(d) and 3(e). With further increasing stress induced by indentation, ITBs formed close to the node to release the stress, emitting a partial from the node due to its high energy stored. A stacking fault was generated along a {111} plane, nucleating and growing a twin by the successive emission of the partials from the node with increasing tress by indentation. This process repeats until the formation of fivefold deformation twins, as depicted in Figs 2(e,f) and 3(f).

There is a 7.35° misfit for an ideal fivefold deformation twin, and therefore stress exists in a FT, leading to the nonuniform angles formed in a fivefold deformation twin, as shown in the insets of Fig. 1(b). This is consistent with previous results^9^,^13^,^19^. Nevertheless, nanoindentation is used in this work to form FTs, which is different from previous reports, in which annealing^13^ and high-pressure torsion^9^,^19^ are employed. Moreover, FTs generates in a single crystal of a ternary alloy in this study, which is distinct from previous pure nanocrystalline Cu^9^,^13^,^19^. The FTs store high stress and energy, especially in their central nodes, resisting effectively for the deformation. Furthermore, they can emit successive partial dislocations from their central nodes during the release of stress, making them grow bigger after loading, as seen in all the three FTs in Fig. 1. The release of stress takes place in a single crystal of a ternary alloy under nanoindentation in this study, which is different from previous findings, in which it happens in small particles of single elements^20^.

In this study, fivefold deformation twinning is performed by MD simulations in a single crystal of an fcc alloy. Fivefold deformation twins are observed in the single crystal under indentation. The formation mechanism of fivefold deformation twins is different from previous reports, in which the partial dislocations are emitted from the GBs. In this work, the partial dislocation is emitted from the ITBs with high energy. After the emission of the partial, a stacking fault is generated along a {111} plane, resulting in the nucleating and growing of a twin by the successive emission of the partials. A node is formed at the intersecting center of the four different slip planes. With increasing stress by the indentation, ITBs come into being close to the node, emitting a partial dislocation from the node. This makes a stacking fault generate along a {111} plane, nucleating and growing of a twin via the successive emission of the partials. The course continues, generating a fivefold deformation twin. The prediction of MD simulations sheds light on the preparation of fivefold deformation twins in single crystals and coarse-grained metals by plastic deformation.

## Methods Summary

An fcc ternary Ni superalloy named C-2000^®^ is widely used in the corrosion-resistant engineering^18^. The nominal composition of C-2000^®^ is 23Cr, 16Mo, 1.6Cu, 2Co*, 3Fe*, 0.5Mg, 0.5Al*, 0.08Si, 0.01 C (wt.%), and Ni balance (* meaning the maximum). The C-2000^®^ was selected as the fcc ternary alloy in MD simulations with chemical compositions of 61Ni, 23Cr and 16Mo (wt.%). Embedded atom method (EAM) is an effective approach to well describe the interatomic forces^19^,^20^,^21^ for a metal. The total energy E in a metal is presented^21^,


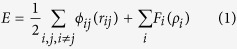


where 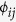 is the pair energy between atoms i and j separated by a distance *r*_*ij*_, *F*_*i*_ the embedding energy, and *ρ*_*i*_ the electron density. The electron density is calculated^22^,^23^,


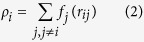


with *f*_*j*_(*r*_*ij*_) the electron density at the site of atom i arising from atom j at a distance *r*_*ij*_ away.

For a single elemental metal, the EAM potential is composed of three functions: pair energy, electron density and embedding energy. However, for an alloy, the EAM potential consists of not only the three functions for each of the constituent elements, but also the pair energy between different elements. Thus, the EAM potential of a single elemental metal can not be directly used in an alloy. Nevertheless, by normalizing the EAM potentials of constituent elements in an alloy, a generalized EAM potential for an alloy is constructed^21^. The generalized pair potentials of an alloy is expressed^23^,


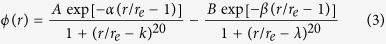


where *r*_*e*_ is the equilibrium spacing between nearest neighbors, *A, B*, α and β are four adjustable parameters, and *k* and *λ* are two additional parameters for the cutoff. The electron density function is addressed^23^,


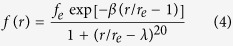


The pair potential between different elements *a* and *b* is written^23^,


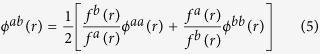


To smooth the embedding energy over a wide range of electron density, the embedding energy functions are divided into three parts to match values at their junctions^23^,


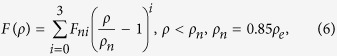



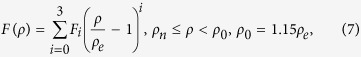



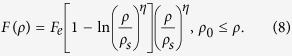


The generalized EAM potential of the C-2000^®^ is constructed according to eight equations, and the parameters needed to define the EAM model are listed elsewhere^21^,^22^,^23^,^24^.

A single crystal of MD model was constructed for the C-2000^®^. The single crystal was 42 nm in length, 1.5 nm in width, and 30 nm in height. The orientations of length and height were [111] and [11-2], respectively. A cylinder was used as the indenter with a tip radius of 15 nm. Displacement-controlled mode was imposed on the single crystal during indentation process. The indentation speed was 20 m/s, and the maximum indentation depth was 10 nm. Periodical boundary conditions were applied on the length and width directions, and shrink-wrapped boundary conditions were implemented on height direction. Prior to indentation on [11-2] orientation, the single crystal was relaxed by an isothermal-isobaric thermostat (NPT) at 300 K for 100 picoseconds (ps). A time step of 1 femtosecond (fs) was used during the indentation. A dwelling time was 5 ps after loading. Common neighbor analysis (CNA) was used to identify the defects induced by indentation. In CNA method, ore red line represents a TB, and multiple red lines stand for staking faults. Green color means perfect atoms, red color for hexagonal close-packed (hcp) atoms, blue color for body-centered cubic (bcc) atoms, and white color for other deformed atoms.

## Additional Information

**How to cite this article:** Zhang, Z. *et al*. Formation mechanism of fivefold deformation twins in a face-centered cubic alloy. *Sci. Rep.* **7**, 45405; doi: 10.1038/srep45405 (2017).

**Publisher's note:** Springer Nature remains neutral with regard to jurisdictional claims in published maps and institutional affiliations.
